# From diagnosis to dialogue: a call for support of older adults with a late autism diagnosis

**DOI:** 10.1093/geront/gnaf181

**Published:** 2025-09-16

**Authors:** Marije Blok, Frans W Jacobs, Arjan C Videler, Hilde M Geurts, Jan Pieter Teunisse

**Affiliations:** Department of Sociology, Faculty of Social Sciences, VU University Amsterdam, The Netherlands; Department of Strategy, Stichting Laurens, Rotterdam, The Netherlands; Research Group ‘Fulfilling Life with Autism’ (Volwaardig Leven met Autisme), HAN University of Applied Sciences, Nijmegen, The Netherlands; PersonaCura, Clinical Center of Excellence for Personality Disorders and Developmental Disorders in Older Adults, Tilburg, The Netherlands; Tranzo, Scientific Centre for Care and Wellbeing of the Tilburg School of Social, Behavioral Sciences of Tilburg University, Tilburg, The Netherlands; Dutch Autism & ADHD Research Center (d’Arc), Brain & Cognition Department of Psychology, University of Amsterdam, The Netherlands; Leo Kannerhuis, Center for Autism, Arnhem and Amsterdam, The Netherlands; Research Group ‘Fulfilling Life with Autism’ (Volwaardig Leven met Autisme), HAN University of Applied Sciences, Nijmegen, The Netherlands; Leo Kannerhuis, Center for Autism, Arnhem and Amsterdam, The Netherlands

**Keywords:** Wellbeing, Psychology of aging, Psychiatry, Autism, Neurodiversity

## Abstract

Although both in autism and gerontological research the attention for aging well with autism is growing, little attention has been paid to the impact of receiving an autism diagnosis in later life. This is unfortunate, as nowadays many of the older adults on the autism spectrum have spent most of their lives being unaware of their autism. Drawing on previous research, our combined personal and professional experiences, and the narratives of eighteen older adults diagnosed later in life (aged 60–77, 50% female), we argue that a late autism diagnosis necessitates a fundamental rethinking of aging, identity, personal relationships, and appropriate support. Effective interventions must be tailored to the specific needs and strengths of this population, ensuring that late-diagnosed older adults with autism receive the recognition, understanding, and tools they require to navigate their lives with confidence and self-awareness. Our findings highlight the need for a conceptual shift in how gerontology understands and addresses autism among older adults. In addition, the narratives of the older adults informed the (co-)creation of a conversational tool designed to help older adults with autism articulate personal boundaries and aspirations. By fostering understanding and dialogue, this initiative extends beyond individual transformation to promote broader societal awareness and support.

## An autism diagnosis in late adulthood: A transformative experience

Receiving an autism diagnosis in late adulthood is a transformative experience that extends far beyond the moment of diagnosis (Lenders et al., 2024; Stagg & Belcher, 2019). It prompts both a re-evaluation of past experiences and a reconsideration of future possibilities, with profound psychological, social, and practical implications. Diagnosed with autism, the first two authors have personally experienced this transformation themselves. In 2019, just prior to his retirement, researcher Jacobs received an autism diagnosis. This had a decisive impact on his life and prompted him to seek therapeutic support. He engaged in psycho-education, extensive reading, and conversations to gain deeper understanding. Feeling the need to share his experiences, Jacobs initially provided family and friends with theoretical knowledge about autism. However, as he realized that theoretical insights did not fully reflect his personal experiences as a father, brother, and friend, he desired to work on a tool that would facilitate meaningful personal conversations about living with autism. Not only for his own use, but also for other older adults with autism. Jacobs initiated this project in close collaboration with researchers and therapists with expertise in the field of aging and autism, designers, and autism organizations. Blok, diagnosed with autism in 2018, at the age of 31, later joined the project, bringing her expertise as a researcher in gerontology focusing on the social and emotional well-being of older people. Next to her role as a researcher, she works as a strategist in a large care organization for older adults in the Netherlands. Our aim was to gather lived experiences regarding a diagnosis in late adulthood, serving both as input for concrete tools with real impact on people’s day-to-day lives as well as to share this important story with a broader audience, both in research and practice. Despite growing academic and societal attention to autism in adulthood, there remains a significant gap in practical support for older adults who received an autism diagnosis in late adulthood.

With this contribution, we call for the development of better resources and support for older adults receiving an autism diagnosis in late adulthood. Drawing on previous research, our combined personal and professional experiences, and the narratives of eighteen older adults diagnosed later in life, we point out that a late autism diagnosis necessitates a fundamental rethinking of aging, identity, personal relationships, and appropriate support. Facilitating this process for older adults requires a fundamental shift in how neurodivergence is understood across multiple domains, including research, health care, and social services. Existing paradigms tend to overlook autism in later life (O’Nions et al., 2023; Winnemuller et al., 2024). In clinical practice, late-life diagnosis is often seen as unnecessary or unhelpful, which contributes to its under-recognition and under-representation. We argue instead that recognizing autism in later life should be framed as a meaningful lens through which individuals can reinterpret their life course and social roles. Beyond closing a life-long quest, diagnosis in later life can open space for renewed dialogue, identity and support. We not only call for this conceptual shift but also offer concrete recommendations.

## Increased attention for a hidden group

Recent years have seen growing awareness, recognition, and research regarding (characteristics of) autism in older generations which enhances our understanding of the lifelong challenges of this group (Heijnen-Kohl et al., 2025; Sonido et al., 2020). This is crucial, as our society increasingly expects older adults to age independently, and this group has particular needs for support in this. Moreover, understanding one’s personal identity is key for promoting mental well-being and self-esteem (Corden et al., 2021; Kiehl et al., 2024), and a diagnosis can help in this process. Also in research—both in the domain of autism and gerontology—the attention for older adults living with autism is growing. However, as much research focuses on aging well with autism (e.g., Brown et al., 2023; Stringfellow et al., 2024), much remains unknown about personal experiences with and the impact of the late autism diagnosis as such.

Note: In this project, we worked together with Dutch older adults, who preferred person-first language. This is not only in line with the preference of the diagnosed researchers in this project, but also with outcomes of previous research in this respect that has shown that the majority of Dutch-speaking adults with autism have a person-first-language preference (Buijsman et al., 2023). As we are aware that this is in contrast to findings from English-speaking countries, we would like to emphasize our respect for both perspectives.

## Self-disclosure when the sense of self is unsettled

In his contribution “Stigma, Stereotypes, and Self-Disclosure: Disability and Empowerment in Older Adults on the Autism Spectrum,” Eliassen (2025) recently calls for the empowerment of older adults with autism in self-disclosing constructively. This would enable them to be authentic and valued members of society, fostering expectations of interdependence as opposed to either complete independence or helpless dependency. A relevant call, as research shows that many older individuals with autism do not receive the understanding and hence the care and support they need (Mason et al., 2021). With our contribution, we build upon this call. We argue the need for a conceptual shift in both research and practice and provide practical recommendations to empower older adults and their social and professional environment. Namely, studies emphasize that if more self-disclosure is asked from older adults than what they would prefer, this could have the opposite effect than intended and can be at the cost of their wellbeing (Derlega et al., 1993). Especially for those with autism, a careful approach is important. In his contribution, Eliassen (2025) elaborates on the paradox that self-disclosure is particularly important for older adults with autism to receive support with, e.g., social vulnerabilities, but due to these social vulnerabilities, self-disclosure is particularly difficult for them. In addition to this paradox, we would like to discuss another paradox, why we would expect self-disclosure to be complicated for this group of older adults. Self-disclosure is about sharing personal information about the self. However, a sense of self is often difficult for people with autism (Cooper et al., 2021). This is true for all individuals receiving an autism diagnosis, let alone those receiving this diagnosis in late adulthood (Nayyar et al., 2025). Research indicates that adults receiving an autism diagnosis in late adulthood often undergo a significant period of identity reconstruction, as they integrate this new understanding into their personal and social narratives. For many, the late diagnosis brings a profound realization that they have unknowingly lived with autism for much of their lives (Huang et al, 2023; Stagg & Belcher, 2019). So promoting self-disclosure among older adults with autism not only asks for purely improving disclosure, but also requires the support in re-discovering the self after a late diagnosis. In other words: what is self-disclosure when the sense of self is unsettled?

## Nothing about us, without us

Our project started with co-author Jacobs’ personal desire to create something meaningful for older adults diagnosed with autism in late adulthood. His lived experience provided a valuable starting point, but from the outset, he was mindful of the adage that “*once you’ve met one autistic person, you’ve met one autistic person*.” In other words, we recognized that developing truly effective tools required not just designing *for*, but more importantly together *with* this group. As a research team, we engaged in ongoing reflexive dialogue to examine how our lived and professional experiences with aging and autism shaped our assumptions, interpretations, and operationalization. Although our personal experiences were valuable, we also recognized the risk of limiting what could emerge from the encounters. Reflexivity was key to maintaining openness throughout the process. Rather than a purely academic data collection process, our approach centered on peer engagement: testing our assumptions, refining our ideas, and co-developing a practical tool together with the target group. Research underscores the significance of lived experience in shaping the understanding of autism (Gillespie-Lynch et al., 2017). Older adults with autism, in particular, have accumulated valuable insights over a lifetime, navigating marginalization, accommodations (or the lack thereof), and support networks (Antonucci et al., 2014). Their expertise is crucial in driving meaningful change at individual, institutional, and societal levels. To explore whether our personal and professional experiences, as well as insights from previous studies, resonated with a broader narrative, we engaged with 18 older autistic adults (aged 60–77, 50% female). These individuals had received an autism diagnosis on average 6 years prior. Seven interviews were conducted orally, and eleven in writing. Although the topics explored in the conversations were partly informed by research literature—particularly on themes such as social connectedness (e.g., Stewart et al., 2024), stigma (e.g., Turnock et al., 2022), and communication (e.g., Nicolaidis et al., 2015)—the overall format was inductive, allowing participants to articulate their experiences in their own wordings. The input of Jacobs, bringing his lived experience of a late diagnosis, contributed to the sensitivity and relevance of the questions. Based on this, the final interview guide included 35 open-ended questions, covering different stages of the participant’s journey, including background (7), the diagnosis (7), life before (5), and after the diagnosis (16). INK social design, involved to co-design a practical tool with the target group, conducted the interviews. The narratives of the 18 older adults with autism highlighted the profound impact of receiving an autism diagnosis later in life and reinforced the urgent need for practical tools to help them express themselves on an interpersonal level. Additionally, their insights directly informed the development of such a tool. Five key themes emerged as essential considerations for creating practical tools for this group: the diagnosis; vulnerabilities; strength; coping mechanisms; and responses of others. By engaging with this community, we were able to move beyond assumptions and ensure that the tool we developed was both relevant and genuinely beneficial to those it was designed to support.

### The diagnosis—a relief and a verdict at the same time

The conversations were in line with our own experiences: receiving a diagnosis in late adulthood brings both relief as well as a verdict, a judgment. For many older adults, the diagnosis finally provided an explanation for past struggles. At the same time, it could feel like a confronting judgment of the life they had lived before. In line with what previous studies emphasize (e.g., Kentrou et al., 2024), many had received incorrect or incomplete diagnoses earlier in life. This had led to misunderstanding in the past and a sense of sadness in the present. For all participants, also the eventual autism diagnosis triggered deep emotions. Several even described it as the most significant moments of their lives. It provided recognition and understanding, making self-acceptance easier:

*Now I understand what is going on with me, it’s easier for me to accept that life has unfolded the way it has. There is finally recognition of why I am so different from others. I was glad to learn about that.*

Others mainly experienced feelings of grief, sadness, and loss. The diagnosis made them reflect on a life in which they had unknowingly navigated the world with autism, often feeling like outsiders and missing opportunities

*I felt grief and sadness that this was not recognized nor discovered my entire life.*

The diagnosis made respondents look back on their lives with autism without knowing, often with a sense of being an outsider. These lives were for many an unsatisfactory effort to participate fully and connect with others.

*I focused so much on others that I no longer knew who I was. I constantly have to be on guard with people. I've been rejected all my life, was never loved, and that hurts.*

Based on these conversations and reflection on our own experiences, we emphasize that although an autism diagnosis in late life can be pivotal for understanding one’s life narrative, it should be regarded as a starting point rather than a solution. Many older adults diagnosed in later life struggle with existential questions regarding their identity and the process of (re)shaping their lives. This underscores the necessity of a mind shift in aging and autism in later life, mainly among health care professionals. Rather than framing the diagnosis as an endpoint, it should be considered as a catalyst for ongoing self-exploration. There is an urgent need for practical tools that can facilitate this process of rediscovery, not only to foster self-acceptance but also to better structure the support and accommodations these individuals require. A diagnosis alone is insufficient; what is needed is a comprehensive structured approach to help older adults navigate this life-changing revelation and find their place in society. Moreover, although we assert that a diagnosis is not a solution in itself, we argue that it is valuable to start the process of diagnosis, as it brings new perspectives. Currently, we see many clinicians hesitate to initiate diagnostic processes for older adults, questioning the potential benefits of it. This insight further supports the need for a paradigm shift in which clinicians routinely consider the diagnostic process not only relevant but essential, even at a high age.

### Vulnerabilities: (mis)understanding the social world

A key vulnerability shared in the interviews was the difficulty of the older adults in understanding and navigating the social world. This often led to feelings of exclusion and loneliness. Many only became aware of the significance of social connections—and their own struggles with autistic traits—after receiving a diagnosis later in life. Looking back, they often saw a lifetime of social disconnection, a persistent effort to belong that never truly succeeded:

*As if it were a play where everyone had learned and practiced their lines, and I was suddenly added without a script.*

Difficulties in recognizing social cues often dated back to childhood. All respondents had struggled with social engagement. Many of them felt excluded, uninteresting, and unattractive to others, not fitting into social circles, despite years of trying:

*I felt like a sort of Martian. With the traits of a human, but I didn’t know how people interact with each other. It took me half a century to figure that out—only a little.*

Due to insufficient social abilities, many respondents experienced minimal (social) self-confidence and high self-criticism. They felt that they were not capable to maintain friendships:

*I am not able to maintain friendships. Never had any friends myself. “Our” friends are friends of my wife. I am champion in ‘chasing friends away’…*

All respondents described their lives until the diagnosis as a struggle. They shared insistent testimonies about loneliness, not feeling understood, nor understanding others:

*My life before the diagnosis was a struggle with life. Stumbling, not understanding anything, depression. Always tired, never enjoying anything. I get emotional very easily, and that’s what’s happening now too.*

The struggles of the older adults in our project are in line with our own personal and professional experiences as well as previous studies on this topic. Literature on self-disclosure and autism (Eliassen, 2025) highlights a critical paradox: individuals with autism often need support in social situations but find it difficult to ask for it in a strategic and socially effective way, due to these particular social struggles. To receive the right support, older autistic adults must be able to articulate their needs. It is essential to support them in formulating and expressing their vulnerabilities in a constructive way, so that they can receive proper support. Although loneliness and social vulnerability in later life increasingly receive attention in gerontological research (e.g., Huang et al. 2025), in its approach (undiagnosed) neurodivergence is often still overlooked. Older adults with autism are, therefore, often misread through an (ageist) lens that fails to capture the nature of their social difficulties—even in an era where person-centered care is increasingly considered important. To change this, paradigms in research and care must evolve to include autism as a valid part of the life course, one that demands specific interpretation, support, language, and recognition. Framing the diagnosis as a point of reorientation rather than closure enables more compassionate and effective support.

### Strength: self-awareness as a strength

Older adults with an autism diagnosis in late adulthood have spent a lifetime feeling different from others. Over the years, they have consciously developed strategies to navigate daily life, making self-awareness one of their key strengths. Several participants emphasized how their deep understanding of themselves had helped them function in a world that often feels unpredictable. One of the participants had even created a personal manual to help others understand their way of thinking and interacting:

*At one point, I made a kind of manual about myself, especially for my colleagues—how to react to me, why I respond in certain ways, etc.*

Next to self-knowledge and self-awareness, our participants found purpose in structured societal roles, such as volunteering or serving on boards. Perseverance was another frequently mentioned strength: many participants described themselves as determined problem-solvers, learning through trial and error and finding alternative routes when faced with setbacks. Recognizing the strengths of older adults with autism is crucial for moving beyond deficit-based models of autism in late life. Instead, a conceptual mindset is needed in which late-diagnosed older adults are not primarily seen as passive recipients of care, but as individuals with valuable insights and adaptive capacities. Practical tools building on self-knowledge—such as guided exercises to map one’s preferences, sensitivities, and strategies—can support older adults in articulating their needs. Framing self-awareness as a strength, rather than a side effect of lifelong struggle, is key to enabling autonomy, better relationships, and meaningful participation in society.

### Coping: credits and costs

Most participants were proficient at putting into words how they cope with challenges and vulnerabilities. Many of them indicated that they had spent their entire lives adapting. Facing both implicit and explicit pressure to conform to socially desirable behavior–at home, at work, and in public—they worked hard to understand the social world. Although this effort allowed them to participate in social situations, the cost was high. Camouflaging their true selves often led to exhaustion, chronic stress, and even burnout:

*I did learn to act socially. Like keeping an eye on myself not talking too much. I’m very alert in social situations. ­Diagnosis leads to awareness. This is positive but at the same time, it makes me aware of the enormous number of stimuli and discomforts. At home, on the street, at work, in the store, in the park, everywhere. These stimuli cause one to constantly operate at the limit of one’s possibilities. People around you have no idea of that.*

Another way participants dealt with (social) challenges is the avoidance of difficult situations, which for many had increased throughout their lives. However, balancing between the desirability of a social life and meeting the need to monitor energy levels, also poses dilemmas:

*I don’t want to appear blunt and uninterested in others, but at the same time I dread birthdays and other parties. I find it hard to find a good balance in that. Usually, I do more than would be good for me and get overstimulated and therefore negative and surly. I would also like more contact with my children, but that takes a lot of energy.*

Several participants found hold in interests and activities. Examples included working in the IT domain, finding structure, clear goals, working independently, or a strong focus on functional contacts. A functional role conferred legitimacy and more distant relationships, as opposed to the vulnerability of closer people.

Even though it is usually not visible to the outside world, the coping mechanisms of older adults with autism cost them a lot of energy. Participants even referred to these mechanisms as survival strategies. Moreover, camouflaging often led to confusion and doubts regarding oneself. Years of adapting to others often blurred the line between their true selves and the roles they had learned to play. In addition, as described earlier, the diagnosis itself also was disruptive and led to confusion about one’s own identity. This insight underscores the urgency of a conceptual shift in the support of older adults: one that moves beyond viewing “well-masked” functioning as success, underestimating the severity of autism, and instead acknowledges the hidden costs of lifelong adaptation. Practical post-diagnostic support must help older adults disentangle their authentic identity from years of coping. By embedding such exploration in structured formats—such as guided reflection tools—older adults are more likely to engage in a constructive way.

### Responses of others: need for knowledge

Many participants elaborated on the public perception of autism to be full of—often false—assumptions. As a result, they often felt either stigmatized or trivialized. Many participants observed a limited knowledge about autism among others. They emphasized that an increase of knowledge in society would be beneficial. According to participants, this lack of knowledge is due to limited frames of reference:

*People notice that I am different, but many people don’t believe I have autism, because they don’t think I match ‘the picture’.*

*It would be good if the outside world would know what autism actually means and especially that it covers a large spectrum.*

Several respondents experienced stigmatization and prejudices regarding their independence or level of functioning. Some even expressed regret at being open about their diagnosis:

*At first, I thought: if I tell at work, they can take it into account, but I only had negative experiences after that. After I was diagnosed, I was treated differently. Stigmatized, pigeonholed. In hindsight I think I should never have told it, should have kept it to myself, but that is also very hard.*

For others, this anticipation made them reluctant to share about their autism diagnosis at forehand:

*I am so afraid that if I say something about my autism too quickly, people will run with it. People have in their heads a number of characteristics that they think belong to autism, and they throw them over you like a blanket: ‘That must be the case with you too’. Stop, that doesn’t have to be true!*

Another response due to limited knowledge, was trivialization. In these cases, the impact of autism was not taken seriously:

*Comments like ‘I sometimes forget things too, I don’t always realize everything too’- or in other words, ‘don’t be silly, everyone has things sometimes.*

The participants’ experiences reflect a deeper conceptual issue: the prevailing frameworks often fail to recognize the nuanced realities of adults with autism, especially in later life. They tend to equate autism with visible dysfunction or early-life diagnoses, leaving little room for more complex or subtle presentations. This not only reinforces stigma but also discourages older adults from sharing their experiences. This particular insight prompted deep reflexive engagement among the researchers with autism in our team. They, too, have faced personal barriers to disclosing aspects of their identity in order to access appropriate support. Although sharing these reflections involved a degree of vulnerability, this moment of insight became pivotal in shaping this contribution. It underscored the importance of embracing our own positionality and taking responsibility for the role we play in challenging the status quo and fostering change. Namely, increased knowledge about the vulnerabilities and impact of autism is needed in society, based on a more heterogeneous population of people with autism. As emphasized in previous literature (e.g., Eliassen, 2025), we believe (older) individuals with autism can play an important role themselves in providing valuable, broader perspectives aimed at combating stigma and stereotypes. Practical tools can support older adults in formulating these perspectives and express themselves in a constructive way so policy and research can benefit from their input. Additionally, as highlighted by Eliassen (2025), self-disclosure is essential for fostering intimacy in personal relationships. However, many older adults hesitate to share personal information with others due to past experiences of stigma or trivialization. When disclosure is met with misunderstanding or dismissal, it discourages further openness about autism-related challenges. Previous research on self-disclosure among older adults shows that intimacy will mainly be improved when it is reciprocal. In other words, if both conversation partners share something about themselves equally (Blok et al., 2022; Reis & Shaver, 1988). A practical tool should, therefore, not only encourage older adults with autism to share their stories but also prompt their conversation partners to open up as well. By fostering balanced, reciprocal exchange of experiences, such a tool could help create meaningful interpersonal connections.

### Translation into a practical tool

In co-creation with eigtheen older adults with an autism diagnosis in late adulthood, we translated the call for support into a practical tool. This tool supports older adults with a late autism diagnosis in (re)discovering and expressing their identity. It encourages them to constructively share vulnerabilities and ask for proper support, and provides a structured framework to build upon the strength of self-knowledge. Finally, it facilitates reciprocal and equal interpersonal conversations.

The tool Wensen *en Grenzen (Aspirations and Personal* Boundaries) fosters meaningful dialogue between (older) adults with autism—either diagnosed or not—and others^1^. The tool consists of 100 question cards, divided into five themes: strengths, personal, looking back, social contact, and future. This set of cards helps adults and others mutually to reflect on their experiences and practice reciprocity with understanding each other in a safe environment. In test sessions, older adults with autism and their partners considered the tool as promising. A couple which was facing major relationship problems shared:

*We are so grateful to you for developing this. Because we were able to ask each other questions that we never asked each other before. We are so happy with it.*

Additionally, we organized a dozen workshops in professional settings in the Netherlands, such as at mental health institutions and the Dutch Association for Autism (NVA). In all cases, both professionals and older adults with autism and their partners considered the set of cards as a very useful tool for conversation.

Our contribution highlights the need for a conceptual shift in how gerontology understands and addresses autism among older adults. This motivated the (co-)creation of a conversational tool designed to help older adults with autism articulate personal boundaries and aspirations. By fostering understanding and dialogue, this initiative extends beyond individual transformation to promote broader societal awareness and support. Improving connections among people with and without autism hopefully results in a positive change at an individual level and, eventually, at a societal level.

## Data Availability

This article does not report scientific data and, therefore, the pre-registration and data availability requirements are not applicable.

## Notes

1
https://www.hogrefe.com/nl/shop/wensen-grenzen-gesprekskaarten-97956.html
